# Effect of Immersion in Quaternary Ammonium Methacryloxy Silane Mixed Monomer on the Mechanical Properties and Antibacterial Activity of a 3D-Printed Urethane Dimethacrylate Denture Base Resin

**DOI:** 10.3390/jfb16120462

**Published:** 2025-12-14

**Authors:** Kun-Min Kim, Yeseul Park, Jimin Kim, Mu-Yeol Cho, Jee-Hwan Kim

**Affiliations:** 1 Department of Prosthodontics, College of Dentistry, Yonsei University, Seoul 03722, Republic of Korea; mingkim0922@naver.com (K.-M.K.); yeseul189@yuhs.ac (Y.P.); jm321@yuhs.ac (J.K.); 2 Apple Tree Institute of Biomedical Science, Apple Tree Medical Foundation, Goyang 10387, Republic of Korea; rkdvkdnj135@naver.com

**Keywords:** 3D printing, denture base resin, quaternary ammonium silane (QAMS), immersion treatment, antibacterial activity, mechanical properties

## Abstract

Denture base resins are susceptible to microbial colonization, and current antibacterial additives often lose effectiveness and may weaken material properties. This study evaluated whether immersion in a quaternary ammonium methacryloxy silane (QAMS)-containing monomer can enhance antibacterial activity without compromising the mechanical properties of digital light processing–printed urethane dimethacrylate denture base resin. Specimens of printed denture base resin were immersed in mixtures of denture base resin and a QAMS-containing monomer at ratios of 10:0 (Control), 7:3 (K3), 5:5 (K5), 3:7 (K7), and 0:10 (K10), followed by post-curing. Flexural strength and modulus were measured by three-point bending, and surface hardness was assessed by Vickers microhardness testing. Antibacterial activity against *Streptococcus mutans* was assessed by inhibition-zone and colony-counting assays. All QAMS-treated groups preserved flexural strength, with a slight reduction in modulus in K5 (*p* < 0.05), while hardness remained unchanged. Antibacterial activity improved in all QAMS-treated groups; K5 and K7 showed the strongest results. Surface analyses using scanning electron microscopy and energy-dispersive X-ray spectroscopy verified formation of a Si-rich modified layer. QAMS immersion followed by post-curing produced a stable, contact-active antibacterial surface without reducing mechanical properties. Among the formulations, K7 (~21 wt% QAMS) provided the most favorable balance of antibacterial activity and mechanical performance.

## 1. Introduction

The oral cavity contains a diverse microbial ecosystem responsible for diseases, including dental caries and periodontal infections. These microorganisms readily form biofilms on dental materials, particularly on resin surfaces [1,2,3,4]. Among dental materials, denture base resins are particularly prone to microbial colonization. Poor oral hygiene and continuous denture use, especially in older individuals, promote bacterial and fungal accumulation, often resulting in chronic infections, such as denture stomatitis [4,5,6,7,8]. Such microbial reservoirs have been associated with aspiration pneumonia, a leading cause of mortality in older adults, as denture wearers exhibit a higher risk of pneumonia than non-wearers [9,10,11,12]. Therefore, controlling microbial adhesion and biofilm formation on denture surfaces is essential for improving the long-term oral and systemic health of denture users.

The rapid transition toward computer-aided design/manufacturing (CAD/CAM) and three-dimensional (3D) printing technologies has revolutionized denture fabrication, offering improved accuracy, efficiency, and prosthesis fit [13,14]. However, stereolithography (SLA) and digital light processing (DLP) systems, which fabricate denture base resins by sequentially curing photopolymer layers only tens of micrometers thick, inherently produce a stepping effect. This layer-by-layer pattern can increase surface roughness (Ra), and values exceeding 0.2 μm have been associated with enhanced initial microbial adhesion [14,15,16,17]. Consequently, 3D-printed denture base resins remain vulnerable to microbial colonization and biofilm formation, similar to their conventionally processed counterparts [18,19].

Recent studies have therefore, explored surface modification strategies aimed at reducing microbial adhesion on denture materials; these approaches mainly involve either altering surface energy and roughness or introducing antibacterial functional groups onto polymer surfaces [17,19,20,21,22,23]. Plasma treatment, co-polymer coating, and graft polymerization can enhance hydrophilicity and reduce early biofilm accumulation; however, their durability often decreases with mechanical wear and routine cleaning [23].

To address these challenges, various surface-modification strategies, such as coating or glazing have been explored [24]. In addition, antimicrobial additives, such as silver compounds and nanoparticles, have been incorporated into denture base materials [24,25,26,27]. However, their antibacterial effects diminish over time owing to leaching or depletion of the active agents, and such additives may induce undesirable effects, including color change and reduced mechanical strength [28,29,30]. These limitations highlight the need for non-leaching antibacterial approaches that can be integrated into 3D-printable resin systems without compromising their mechanical properties.

In this context, increasing attention has been directed toward quaternary ammonium compounds (QAC), a class of cationic antibacterial agents widely investigated in dentistry. QACs exert their antibacterial activity through a contact-killing mechanism in which their positively charged quaternary ammonium groups disrupt negatively charged microbial membranes [31,32,33,34]. This physical mode of action does not promote antibiotic resistance and offers the potential for long-lasting efficacy because the antibacterial component is not depleted or released [35,36]. Numerous efforts have been made to incorporate QAC-based antibacterial functionalities into dental polymers in a non-leaching manner. Quaternary ammonium dimethacrylate (QADM), dimethyl dioctadecyl ammonium bromide (DDAM), dimethylaminohexadecyl methacrylate (DMAHDM), and quaternary ammonium methacryloxy silane (QAMS)-based monomers have been incorporated into methacrylate resins to impart covalently bound, contact-active antibacterial properties [32,37,38,39,40,41]. Among the QAC systems, the QMAS-based compound referred to as “K18” has garnered particular interest owing to its structural characteristics. K18 contains a silane core, an 18-carbon alkyl chain that enhances membrane interaction, and a methacrylate group that enables copolymerization with polymethyl methacrylate (PMMA)-based monomers [42,43]. Once polymerized, K18 becomes covalently bound within the resin network, providing a contact-killing antibacterial effect with inherently low risk of microbial resistance [38,44,45,46,47,48].

Two major strategies have been proposed to functionalize 3D-printed denture base resins with antibacterial agents: incorporation into the resin before printing and post-polymerization surface modification [22,49]. The incorporation method provides uniform distribution but may alter viscosity, polymerization behavior, and mechanical properties [26,38,50]. In contrast, surface coating methods preserve the underlying resin composition but may be limited by surface-layer instability or gradual loss of functional agents under clinical conditions [24,51]. An ideal approach should ensure stable chemical immobilization of the antibacterial agent while maintaining the intrinsic properties of the 3D-printed resin.

This study introduces a surface immersion treatment using a QAMS-containing monomer solution to impart antibacterial functionality to a 3D-printed urethane dimethacrylate (UDMA)-based denture base resin. As residual double bonds remain on the surface of 3D-printed resins, immersion in QAMS-containing monomer allows additional copolymerization and partial immobilization of QAMS molecules upon subsequent post-curing [52]. This study aimed to evaluate the antibacterial activity and mechanical properties of the modified resin, with the null hypothesis that immersion in QAMS-containing monomer would not cause significant differences compared with controls.

## 2. Materials and Methods

**Figure 1:**
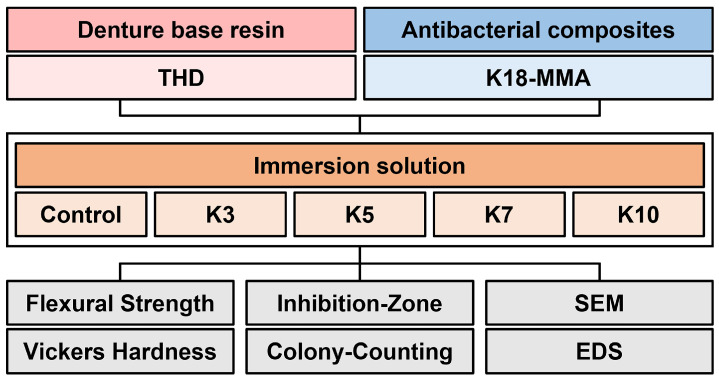
Flowchart of the study.

Figure 1 presents the flow of the experimental procedure.

### 2.1. Specimen Preparation

The denture base resin used in this study was UDMA-based 3D printing material (THD, Graphy, Seoul, Republic of Korea). The antimicrobial agent was K18-functionalized methyl methacrylate (K18-MMA, FiteBac Inc., Marietta, GA, USA), a QAC containing 30 wt% K18 copolymerized with methyl methacrylate (MMA). 

Specimens for the experiment were prepared as follows. All specimens were designed using a CAD software program (TinkerCAD, Autodesk Inc., San Rafael, CA, USA). For the flexural strength test, bar-shaped specimens measuring 64 mm × 10 mm × 3.3 mm were designed as per ISO 20795-1 standards [53]. For the Vickers microhardness evaluation, rectangular specimens with dimensions of approximately 30 mm × 10 mm × 3.3 mm were designed, while for the antibacterial tests, disk-shaped specimens measuring 10 mm × 3 mm were designed. The designed STL files were imported into a 3D printer-specific software (UNIZ Dental 1.5.4.35, UNIZ Technology, San Diego, CA, USA) and positioned on the build platform. Supports were oriented vertically on the broad surface of each specimen, and printing was performed using a digital light processing 3D printer (UNIZ Nbee, UNIZ Technology, San Diego, CA, USA). Following printing, specimens were ultrasonically cleaned in 95% isopropyl alcohol (SH-2240D, Saehan Ultrasonic Co., Seoul, Republic of Korea) for 1 min. Subsequently, the specimens were dried in an oven (DAIHAN Scientific, Gangwon, Republic of Korea) at 60 °C for 5 min.

The formulations for specimen immersion were prepared as follows. The denture base resin and QAMS-containing monomers were weighed according to the specified ratios of 10:0, 7:3, 5:5, 3:7, and 0:10, with the total weight adjusted to 100 g for each group. Photoinitiator was added at 1.5 g for the 0:10 ratio and the corresponding amount for other ratios based on the proportion of the QAMS-containing monomers. The mixtures were then homogenized using a planetary centrifugal mixer (ARE-501, THINKY Mixer Korean Agency, Seoul, Republic of Korea) at 1500 rpm for 5 min.

The dried specimens were immersed in the corresponding mixed monomer solutions prepared for each group and sonicated in an ultrasonic cleaner (SH-2240D, Saehan Ultrasonic Co., Seoul, Republic of Korea) for 3 min. After immersion, the specimens were mounted in a centrifuge (IA-W1, inoq, Gyeonggi, Republic of Korea) and spun at 600 rpm for 1 min to remove excess solution. Finally, a single post-curing cycle was performed in a curing unit (Cure-M 102H, Sona Global Co., Ltd., Seoul, Republic of Korea) under a nitrogen atmosphere at an ultraviolet (UV) energy density of 700 J/cm^2^ for 20 min.

For mechanical property evaluation, 12 bar-shaped specimens per group were prepared for flexural strength testing, and 5 rectangular specimens per group were prepared for Vickers hardness assessment. Additionally, 22 disk-shaped specimens were fabricated per group for antibacterial evaluation, of which 10 were used for the inhibition-zone test and 12 for the colony-counting test. The experimental groups were designated as control, K3, K5, K7, and K10 based on the weight ratio of the QAMS-containing monomers in the immersion solution (Table 1).

### 2.2. Flexural Strength and Flexural Modulus

Mechanical performance was assessed using three-point flexural strength testing. After storage in distilled water at 37 °C for 50 h, each specimen was subjected to testing using a universal testing machine (3366 Series, Instron Engineering, Norwood, MA, USA). The specimens were positioned on a jig with a support span of 50 mm, and the load was applied at a crosshead speed of 5.0 mm/min until fracture occurred. The maximum fracture load was recorded in newtons (N), and the flexural strength (*σ*, MPa) was calculated using the following equation:

(1) $$\sigma =\frac{3FL}{2wh^{2}}.$$

The flexural modulus (E, MPa) was determined from the load–deflection data obtained during the test using the following equation:

(2) $$E=\frac{FL^{3}}{4{wh}^{3}d},$$

where

*F* = Fracture load (N);

*L* = Span length (distance between the centers of the supporting pins, mm);

*w* = Specimen width (mm);

*h* = Specimen height (mm);

*d* = Deflection of the specimen at the fracture point (mm).

### 2.3. Vickers Hardness Test

Vickers hardness was evaluated to assess the surface mechanical properties of the QAMS-treated denture base resin. For each group, five specimens were examined, and three indentations were made per specimen. Measurements were performed using a Vickers hardness tester (MMT-X7, Matsuzawa, Kyoto, Japan) under a load of 500 gf with a 15 s dwell time. The mean value of the three indentations was used for statistical analysis.

Vickers hardness (HV) was calculated as follows:

(3) $$Hv=\kappa \frac{P}{d^{2}}.$$

*κ* = 1.8544;

*P* = Load (kgf);

*d* = Mean diagonal of indentation (mm).

### 2.4. Antibacterial Activity Assay

The antibacterial effect of the specimens against *S. mutans* was evaluated using the inhibition-zone test and colony-counting test. The bacterial strain used in this study, *S. mutans* ATCC 25175, was obtained from the Biological Resource Center of the Korea Research Institute of Bioscience and Biotechnology (Daejeon, Republic of Korea). The strain was cryopreserved at −80 °C in a deep freezer using 10% glycerol as a cryoprotectant. Before each experiment, the frozen strain was thawed and pre-cultured in brain heart infusion (BHI; MB cell, Los Angeles, CA, USA) broth at 37 °C under 10% CO_2_ conditions, and bacterial cells in the logarithmic growth phase were used for testing. All experimental procedures were conducted under a biosafety cabinet to maintain aseptic conditions.

#### 2.4.1. Inhibition Zone Test

In the inhibition zone test, 100 µL of *S. mutans*, which had been pre-cultured in BHI media and adjusted to a concentration of 10^5^ colony-forming units (CFU)/mL, was spread on BHI agar plates using sterile cotton swabs [54]. The specimens were prepared and aseptically placed on the surface of the inoculated plates. The plates were then incubated at 37 °C for 48 h until a uniform bacterial lawn was observed. The inhibition zones were evaluated by measuring their radius, defined as the distance from the center of each zone to its outer edge.

#### 2.4.2. Colony-Counting Test

In the colony-counting test, 100 µL of *S. mutans*, which had been pre-cultured in BHI media and adjusted to a concentration of 10^5^ CFU/mL, was used as the initial bacterial suspension. A 0.5 mL aliquot of this bacterial suspension was added to 5 mL round-bottom tubes containing disk-shaped specimens. For the negative control group, a 0.5 mL aliquot of the bacterial suspension was added to tubes without any specimens. All tubes were incubated at 37 °C for 24 h, after which the cultures were serially diluted in distilled water in seven 10-fold steps up to 10^−7^ for all samples. Aliquots (100 µL) from each dilution were then plated on BHI agar plates, and the CFUs were counted to evaluate the bacterial growth inhibitory effects of the specimens. The negative control group, which did not contain any specimens, was used to account for background bacterial growth. The colony count from the negative control group was also recorded for comparison.

### 2.5. SEM, Energy-Dispersive X-Ray Spectroscopy (EDS)

The surface and cross-sectional morphologies of the specimens were analyzed using field-emission scanning electron microscopy (FE-SEM; JSM-7800F, JEOL Ltd., Seoul, Republic of Korea) to assess microstructural changes following immersion in the QAMS mixed monomer and post-curing. Representative specimens from the control and K7 groups were fractured in liquid nitrogen, sectioned, ultrasonically cleaned in 70% isopropyl alcohol for 1 min, air-dried, and sputter-coated with a 100 nm platinum layer to prevent charging. Observations were performed under high vacuum at 10–15 kV with a working distance of ~10 mm. SEM images were obtained at ×500 and ×2000 magnifications to evaluate surface continuity and the morphology of the modified surface layer. Elemental composition and Si distribution, originating from QAMS, were examined using EDS integrated with the FE-SEM. Elemental mapping and line-scan analyses were performed under 15 kV to assess Si localization within the modified surface layer.

### 2.6. Statistical Analysis

All statistical analyses were performed using statistical software (SPSS version 27.0; SPSS Inc., Chicago, IL, USA). The normality of the data was examined using both the Kolmogorov–Smirnov and Shapiro–Wilk tests. Data for inhibition zone and colony counting were normally distributed and analyzed using one-way analysis of variance followed by Bonferroni post hoc comparisons. In contrast, flexural strength, flexural modulus, and Vickers hardness data were not normally distributed and were therefore analyzed using a non-parametric Kruskal–Wallis test. A significance level of α = 0.05 was adopted for all statistical tests.

## 3. Results

### 3.1. Analysis of Mechanical Properties

#### 3.1.1. Flexural Strength and Flexural Modulus

In the three-point flexural test performed to evaluate the mechanical properties, all groups containing QAMS showed a decreasing trend in flexural strength compared with the control group (66.34 ± 7.31 MPa); however, the differences were not statistically significant. The K5 group (59.08 ± 6.34 MPa) exhibited the lowest mean flexural strength among the QAMS-containing groups (Table 2). 

The mean flexural modulus values of the 3D-printed denture base resin specimens are presented in Table 2. The control group exhibited the highest modulus (1.98 ± 0.21 GPa), which was significantly greater than that of the K5 group (1.81 ± 0.13 GPa) (*p* < 0.05). Overall, the incorporation of QAMS did not result in a consistent concentration-dependent trend in flexural modulus, although a slight reduction was observed in the K5 group.

#### 3.1.2. Vickers Hardness

In the Vickers hardness evaluation, all QAMS-treated groups exhibited values comparable to the control group, and no statistically significant differences were detected among the five formulations (*p* > 0.05). These findings indicate that immersion in QAMS-containing monomer solutions did not alter the surface microhardness of the 3D-printed denture base resin (Table 3).

### 3.2. Analysis of Antibacterial Properties

#### 3.2.1. Inhibition-Zone Test

**Figure 2:**
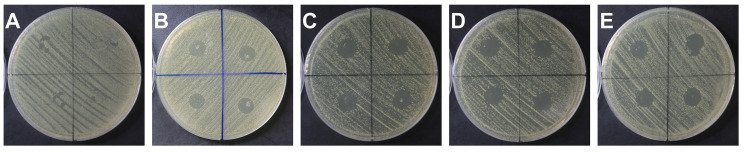
Representative results of the inhibition-zone test of *S. mutans* according to the QAMS concentration in the immersion solution. From left to right: (**A**) Control, (**B**) K3, (**C**) K5, (**D**) K7, and (**E**) K10 groups.

Representative inhibition-zone images for each group are shown in Figure 2, and quantitative results are presented in Table 3. All groups containing QAMS exhibited antibacterial activity against *S. mutans* compared with the control group. The K5 group (12.81 ± 0.25 mm) demonstrated the widest inhibition-zone among all groups; however, the differences were not statistically significant compared with the K7 (12.66 ± 0.34 mm). The K3 group (10.78 ± 0.46 mm) exhibited the narrowest inhibition zone among all groups (Figure 2, Table 4).

#### 3.2.2. Colony-Counting Test

**Figure 3:**
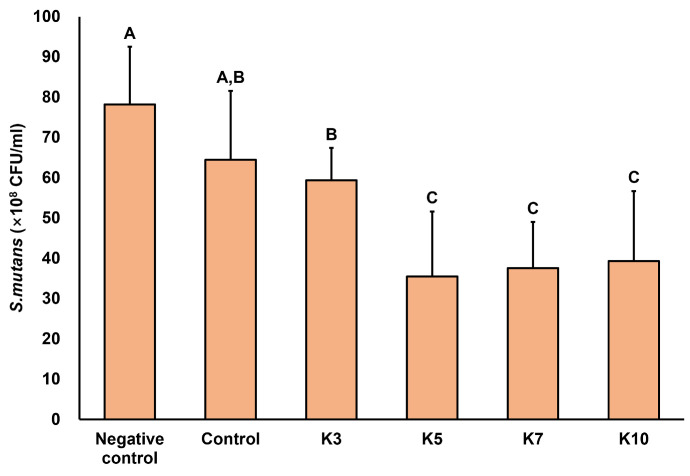
Viable bacterial counts (10^−7^ CFU) were obtained from 3D-printed denture base specimens. Different superscript letters indicate statistically significant differences among groups (*p* < 0.05).

Colony-counting test results are shown in Figure 3. All groups containing QAMS exhibited a significant reduction in CFU compared with the negative control group (*p* < 0.05). Although the K3 group showed a lower CFU count than the control, the difference was not statistically significant. The K5, K7, and K10 groups exhibited significantly lower CFU counts than the other groups, with no significant differences among the K5, K7, and K10 groups (Figure 3).

### 3.3. SEM, EDS

**Figure 4:**
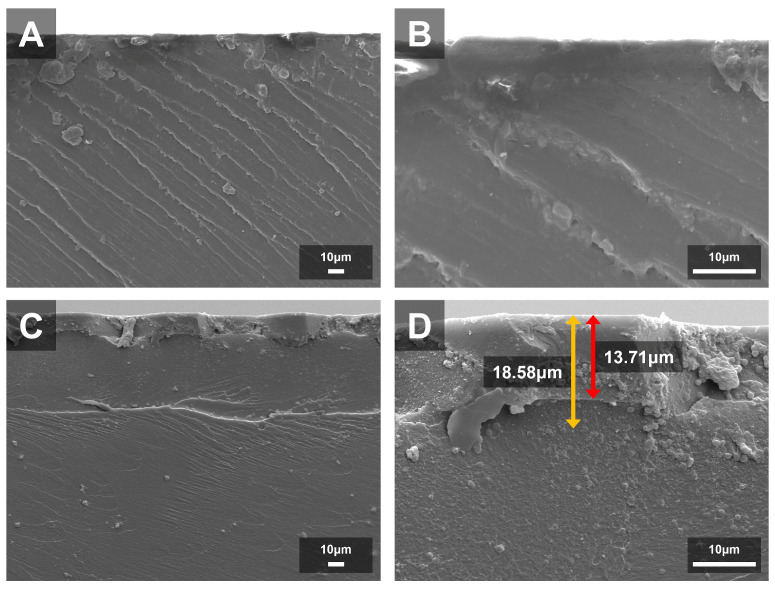
Cross-sectional SEM micrographs of the control and K7 group specimens at different magnifications. (**A**,**B**) Control specimen observed at ×500 and ×2000 magnifications, showing a smooth, homogeneous, and dense matrix without any surface layer. (**C**,**D**) K7 specimen observed at 500× and 2000× magnifications, revealing a distinct surface layer containing fine granular structures. The red arrow indicates the thickness of the surface layer, and the yellow arrow indicates the thickness of the Si-rich layer.

K7 was selected for detailed SEM and EDS characterization because it demonstrated the most favorable combination of antibacterial efficacy and preserved mechanical properties, making it an appropriate representative for assessing QAMS-mediated surface modification. SEM and EDS analyses revealed distinct surface and compositional differences between the control and K7 groups. The control exhibited a smooth, homogeneous surface and a uniform cross-sectional matrix with no detectable Si, confirming the absence of surface modification. In contrast, the K7 specimen displayed a continuous surface layer containing fine granular structures, approximately 13.7 µm in thickness, along with a marked Si enrichment extending to a depth of about 18.6 µm (Figure 4).

**Figure 5:**
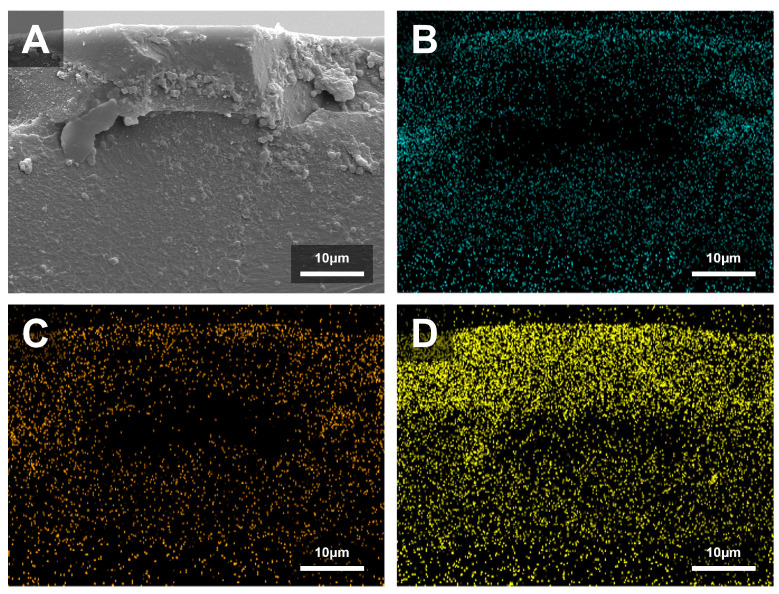
(**A**) Cross-sectional SEM micrograph of K7 group specimen at 2000× magnification. Elemental mapping of the K7 displaying Si ((**D**); yellow) concentrated near the surface, while O ((**B**); magenta) and C ((**C**); cyan) dominate the bulk matrix.

**Figure 6:**
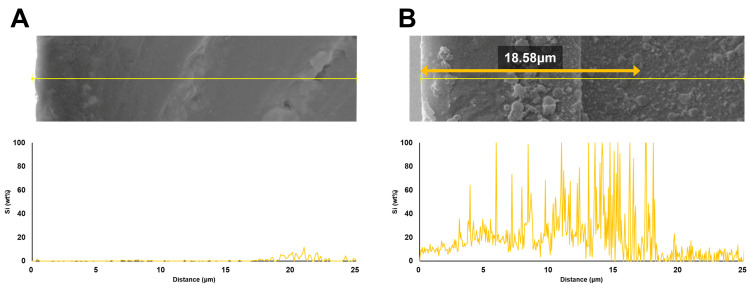
EDS line-scan profiles showing negligible Si signal in the control (**A**) and a sharp Si enrichment at the surface of the K7 specimen (**B**). The yellow arrow indicates the thickness of the Si-rich layer.

Elemental mapping further confirmed that Si was predominantly distributed along the surface layer, whereas C and O were more evenly distributed within the bulk matrix (Figure 5). The quantitative results from EDS line-scan analyses (Figure 6) are summarized in Table 5. The control specimen showed an average composition of 88.25 wt% C, 11.57 wt% O, and 0.18 wt% Si, whereas the K7 specimen exhibited 67.70 wt% C, 20.04 wt% O, and 12.26 wt% Si. These findings confirm that Si was markedly enriched at the surface of the treated specimens compared with the control, verifying the successful formation of a QAMS-derived surface layer through immersion and post-curing.

## 4. Discussion

The null hypothesis proposed that immersion of 3D-printed denture base resin in a QAMS solution, would not yield significant differences from the untreated control. However, a significant enhancement in antibacterial activity was observed in QAMS-treated groups, whereas flexural strength and Vickers hardness remained statistically unchanged, and flexural modulus decreased only in the K5 group. Thus, the null hypothesis was rejected for antibacterial activity and partially rejected for mechanical properties.

The preservation of flexural strength across all QAMS-treated groups suggests that the immersion treatment altered surface properties while maintaining internal matrix integrity. As the immersion process allows QAMS to interact primarily with residual double bonds or free radicals on the surface, penetration into the highly cross-linked UDMA and dimethacrylate network of the denture base resin is likely limited [55,56,57]. This surface-localized modification explains the stable mechanical performance, unlike previous reports where direct QAMS incorporation reduced tensile strength by disrupting cross-linking [38]. Consistent with this interpretation, Vickers hardness values showed no significant differences among groups, indicating that the surface treatment did not adversely affect the micro-scale mechanical behavior of the resin. Together with flexural data, these results demonstrate that the QAMS modification maintained both surface and bulk mechanical performance within clinically relevant conditions.

SEM analysis revealed a smooth and continuous modified layer with fine granular structures, indicating that the immersion process may have induced partial surface re-polymerization or reorganization of residual monomers. Complementary EDS line-scan data showed that Si—originating from the QAMS core—was enriched within this layer and extended to a depth of approximately 18.6 µm, slightly deeper than the ~13.7 µm surface layer identified in SEM. Although Si levels declined sharply beyond this region, low residual signals remained higher than in the control, suggesting limited interaction or infiltration below the primary modified zone.

These combined observations support the interpretation that QAMS functionalization was largely confined to the near-surface region, though subtle subsurface effects cannot be excluded. However, the extent of subsurface change cannot be fully determined from SEM and EDS alone, and the absence of quantitative roughness measurements further limits definitive interpretation. Future studies incorporating profilometric or optical roughness analysis and higher-resolution depth-profiling techniques will be necessary to clarify the structural impact of the treatment and prevent overinterpretation of elemental distribution.

The reduction in flexural modulus observed in K5 group (1.81 ± 0.13 GPa) compared with the control (1.98 ± 0.21 GPa) may be attributed to increased hydrophilicity introduced by the quaternary ammonium group (–N^+^(CH_3_)_3_), which enhances water sorption and plasticizes the resin matrix [58]. Water-induced swelling and reduced intermolecular interactions weaken stiffness and hardness [59,60]. Similar tendencies have been reported for QAMS–PMMA systems, where as little as 2 wt% QAMS reduced flexural modulus due to the plasticizing effect of hydrophilic functional groups [45]. Residual monomers near the surface may also have interacted with monomers containing QAMS during immersion, further softening the matrix [61]. The distinct modulus reduction at the 1:1 ratio suggests a phase-inversion phenomenon between THD and K18-MMA—commonly seen in polymer blends with equal proportions—potentially inducing localized heterogeneity and partial structural destabilization [62,63].

Interestingly, the K7 and K10 groups did not exhibit further deterioration of flexural strength or modulus compared with K5, despite their higher nominal QAMS content. Similar non-linear behavior has been reported for QAMS-containing acrylic resins, where an initial decrease in flexural properties was not exacerbated by further increases in QAMS concentration once a threshold was reached [38,45,46]. This pattern suggests that the actual amount and distribution of QAMS within the modified layer do not scale linearly with the concentration of the immersion solution, and that diffusion limits, saturation, and microstructural organization may play a greater role than nominal loading alone [33,39].

The influence of post-curing must also be considered. Although ultraviolet curing (20 min, 700 J/cm^2^) was applied to polymerize residual monomers containing QAMS, partial light scattering or absorption by the surface monomer layer may have reduced polymerization efficiency, leading to incomplete conversion and localized heterogeneity [64,65,66]. Despite this, the QAMS-modified groups retained acceptable mechanical stability for denture base use, with values comparable to other reported 3D-printed resins [67].

Antibacterial evaluation demonstrated that QAMS-immersed specimens strongly inhibited *S*. *mutans* growth in both inhibition-zone and colony-counting assays. The K5 and K7 groups showed the highest antibacterial activity, indicating a threshold QAMS concentration of ~15 wt% is needed for uniform and continuous antibacterial layer on the surface [35,58,68]. Below this level, discontinuous coverage and insufficient charge density may limit contact-killing efficiency. These findings align with prior QAMS–PMMA data showing enhanced activity beyond a critical quaternary ammonium loading [46].

The antibacterial mechanism follows the well-established contact-killing model of QAMS materials, where cationic groups disrupt negatively charged bacterial membranes, impairing adhesion and viability [44,45]. Although QAMS-modified surfaces may not completely prevent bacterial attachment, they substantially reduce viable cell counts within biofilms and weaken structural stability, leading to detachment under fluid conditions. The current findings align with this mechanism, confirming that a chemically immobilized QAMS layer can impart effective, non-leaching antibacterial activity to the resin surface without the need for leachable components.

Slight bacterial reduction in controls likely reflects the intrinsic hydrophobicity and low surface energy of the 3D printing resin [69]. In this study, the specimens were subjected to EO gas sterilization before antibacterial testing; however, EO treatment does not remove or neutralize residual monomers. Therefore, residual unpolymerized monomers may exert nonspecific cytotoxic effects on bacterial membranes, contributing to reduced viability [55,70]. Nevertheless, the magnitude of antibacterial enhancement in QAMS-treated groups far exceeded such baseline effects, supporting the conclusion that the observed activity primarily originates from the quaternary ammonium modification.

While QACs are known for potent antibacterial effects, potential cytotoxicity has been associated with their leachable forms [46]. Recent reviews on QAM-based dental materials have reported that when QAMs are polymerized into the resin matrix and not allowed to leach, cytotoxicity remains low while long-term antibacterial function is maintained [34,68,71]. Some studies have reported cytotoxic responses in DMAHDM–UDMA systems, particularly at higher concentrations or when residual free monomers remain [72]. However, the cytotoxicity of QAMs is known to be highly dependent on concentration and curing conditions, and polymer-immobilized QAMs generally exhibit reduced toxicity compared with their leachable forms [36]. As cytocompatibility was not evaluated in the present study, dedicated biological testing will be necessary in future work to confirm the safety of the QAMS-modified surface.

*Streptococcus mutans* was selected as a representative microorganism because of its established role in dental biofilm initiation and its association with denture-related infections and aspiration pneumonia. In addition, the lactic acid and carbohydrate metabolites produced by *S. mutans* serve as key nutritional sources for *C. albicans*, a major pathogen implicated in denture-associated complications [7,73,74,75]. Although the antibacterial results were confined to a single bacterial species, previous studies have shown that QAMS-functionalized resins exhibit broad-spectrum antimicrobial activity against *C. albicans*, *S. sanguinis*, and *S. aureus* [35,38,44,45,46,48]. While a surface-immobilized QAMS layer is likely to affect fungal and multi-species colonization, this requires confirmation using biofilm-based models. Because this study focused on the direct effects of QAMS immersion on material properties and antibacterial activity, saliva-mimicking or dynamically simulated oral conditions were not included. Future work incorporating such environments will be necessary to validate the clinical relevance and long-term stability of the antibacterial response.

The antibiofilm effect observed with the QAMS-based monomer treatment suggests potential clinical value, as denture-associated biofilms are major contributors to denture stomatitis and may also serve as reservoirs for respiratory pathogens in older adults [8,11]. Recent systematic reviews have confirmed these associations, and evidence from geriatric trials indicates that improving oral and denture hygiene can reduce pneumonia incidence [8,11,12]. In this context, prior studies of biofilm-targeted interventions—such as daily chlorhexidine rinsing—demonstrate that relative reductions of approximately 40–50% in the rate of pneumonia are considered clinically meaningful [76,77]. If comparable reductions in denture biofilm or stomatitis were achieved clinically, QAMS-based surface modification could provide a favorable balance between efficacy and cost. In vivo studies should verify these effects and compare immersion and bulk-modified QAMS approaches, alongside economic analyses to determine their practical feasibility.

Further studies should include comprehensive surface characterization, cytotoxicity assessment, and evaluation of the durability of the antibacterial effect under conditions that more closely simulate clinical use, including routine cleaning, mechanical wear, and prolonged intraoral exposure. In addition, incorporating biofilm-based models in future antibacterial testing would provide stronger evidence of efficacy under biologically relevant conditions.

## 5. Conclusions

The following conclusions can be drawn within the limitations of this in vitro study. The QAMS immersion method exhibited antibacterial activity against *S. mutans*. Among the tested groups, K7 showed the greatest antibacterial efficacy, while maintaining its mechanical properties. Overall, QAMS immersion provides a simple and effective surface-modification approach for imparting antibacterial functionality to 3D-printed denture base materials, while maintaining acceptable mechanical performance with only minor variations observed in specific properties.

## Figures and Tables

**Table 1:** Composition of Immersion Solutions Based on Weight Ratios.

Group Name	THD (%)	K18-MMA (%)
Control	100	0
K3	70	30
K5	50	50
K7	30	70
K10	0	100

**Table 2:** Flexural strength (MPa) and flexural modulus (GPa) of 3D-printed denture base resin specimens immersed in QAMS solutions with different ratios.

Group Name	Flexural Strength (MPa)	Flexural Modulus (GPa)
Control	66.34 ± 7.31 ^A^	1.98 ± 0.21 ^A^
K3	60.66 ± 4.36 ^A^	1.86 ± 0.07 ^AB^
K5	59.08 ± 6.34 ^A^	1.81 ± 0.13 ^B^
K7	61.30 ± 11.33 ^A^	1.97 ± 0.09 ^AB^
K10	63.73 ± 5.51 ^A^	1.97 ± 0.13 ^AB^

Data are expressed as mean ± standard deviation (SD). Different superscript letters indicate statistically significant differences among groups (*p* < 0.05).

**Table 3:** Vickers hardness of 3D-printed denture base resin specimens immersed in QAMS solutions of different ratios.

Group Name	Vickers Hardness (HV)
Control	20.40 ± 4.39 ^A^
K3	19.51 ± 0.82 ^A^
K5	19.58 ± 1.48 ^A^
K7	20.02 ± 1.41 ^A^
K10	19.51 ± 1.06 ^A^

Data are expressed as mean ± standard deviation (SD). Same superscript letters indicate no statistically significant differences among groups (*p* > 0.05).

**Table 4:** Inhibition-zone diameters (mm) of *S. mutans* for 3D-printed denture base resin specimens immersed in QAMS solutions with different ratios.

Group Name	Mean ± SD (mm)
K3	10.78 ± 0.46 ^C^
K5	12.81 ± 0.25 ^A^
K7	12.66 ± 0.34 ^AB^
K10	12.15 ± 0.64 ^B^

Data are expressed as mean ± standard deviation (SD). Different superscript letters indicate statistically significant differences among groups (*p* < 0.05).

**Table 5:** Energy-dispersive X-ray spectroscopy line-scan data (wt%).

Elemental Composition (wt%)	Control (%)	K7 (%)
C	88.25	67.70
O	11.57	20.04
Si	0.18	12.26
Total	100.00	100.00

## Data Availability

The original contributions presented in this study are included in the article. Further inquiries can be directed to the corresponding author.

## Abbreviations

The following abbreviations are used in this manuscript:

BHI	Brain heart infusion
CFU	Colony-forming units
EDS	Energy-dispersive X-ray spectroscopy
QAC	Quaternary ammonium compounds
QAMS	Quaternary ammonium methacryloxy silane
SD	Standard deviation
CAD	Computer-aided design
CAM	Computer-aided manufacturing
MMA	Methacrylate
SEM	Scanning Electron Microscope

## References

[1] Kreve S., Dos Reis A.C. (2021). Bacterial adhesion to biomaterials: What regulates this attachment? A review. Jpn. Dent. Sci. Rev..

[2] Maier T. (2023). Oral microbiome in health and disease: Maintaining a healthy, balanced ecosystem and reversing dysbiosis. Microorganisms.

[3] Wade W.G. (2013). The oral microbiome in health and disease. Pharmacol. Res..

[4] Teixeira A.B.V., Valente M.L.d.C., Sessa J.P.N., Gubitoso B., Schiavon M.A., Dos Reis A.C. (2023). Adhesion of biofilm, surface characteristics, and mechanical properties of antimicrobial denture base resin. J. Adv. Prosthodont..

[5] Gendreau L., Loewy Z.G. (2011). Epidemiology and etiology of denture stomatitis. J. Prosthodont..

[6] Le Bars P., Kouadio A.A., Amouriq Y., Bodic F., Blery P., Bandiaky O.N. (2023). Different polymers for the base of removable dentures? Part II: A narrative review of the dynamics of microbial plaque formation on dentures. Polymer.

[7] Khoury Z.H., Vila T., Puthran T.R., Sultan A.S., Montelongo-Jauregui D., Melo M.A.S., Jabra-Rizk M.A. (2020). The role of *Candida albicans* secreted polysaccharides in augmenting *Streptococcus mutans* adherence and mixed biofilm formation: In vitro and in vivo Studies. Front. Microbiol..

[8] Perić M., Miličić B., Pfićer J.K., Živković R., Arsenijević V.A. (2024). A Systematic Review of Denture Stomatitis: Predisposing Factors, Clinical Features, Etiology, and Global *Candida* spp. Distribution. J. Fungi.

[9] Alzamil H., Wu T.T., van Wijngaarden E., Mendoza M., Malmstrom H., Fiscella K., Kopycka-Kedzierawski D.T., Billings R.J., Xiao J. (2021). Removable denture wearing as a risk predictor for pneumonia incidence and time to event in older adults. JDR Clin. Transl. Res..

[10] Nakajima M., Umezaki Y., Takeda S., Yamaguchi M., Suzuki N., Yoneda M., Hirofuji T., Sekitani H., Yamashita Y., Morita H. (2019). Association between oral candidiasis and bacterial pneumonia: A retrospective study. Oral. Dis..

[11] Lim T.W., Li K.Y., Burrow M.F., McGrath C. (2023). Prevalence of respiratory pathogens colonizing on removable dental prostheses in healthy older adults: A systematic review and meta-analysis. J. Prosthodont..

[12] Sjogren P., Nilsson E., Forsell M., Johansson O., Hoogstraate J. (2008). A systematic review of the preventive effect of oral hygiene on pneumonia and respiratory tract infection in elderly people in hospitals and nursing homes: Effect estimates and methodological quality of randomized controlled trials. J. Am. Geriatr. Soc..

[13] Dimitrova M., Corsalini M., Kazakova R., Vlahova A., Chuchulska B., Barile G., Capodiferro S., Kazakov S. (2022). Comparison between conventional PMMA and 3D printed resins for denture bases: A narrative review. J. Compos. Sci..

[14] Srinivasan M., Kamnoedboon P., McKenna G., Angst L., Schimmel M., Ozcan M., Muller F. (2021). CAD-CAM removable complete dentures: A systematic review and meta-analysis of trueness of fit, biocompatibility, mechanical properties, surface characteristics, color stability, time-cost analysis, clinical and patient-reported outcomes. J. Dent..

[15] Alqarawi F.K., Gad M.M. (2024). Tendency of microbial adhesion to denture base resins: A systematic review. Front. Oral Health.

[16] Choi J.J.E., Uy C.E., Ramani R.S., Waddell J.N. (2020). Evaluation of surface roughness, hardness and elastic modulus of nanoparticle containing light-polymerized denture glaze materials. J. Mech. Behav. Biomed. Mater..

[17] Kraemer Fernandez P., Unkovskiy A., Benkendorff V., Klink A., Spintzyk S. (2020). Surface characteristics of milled and 3D printed denture base materials following polishing and coating: An in-vitro study. Materials.

[18] da Silva M.D.D., Nunes T.S.B.S., Viotto H.E.D.C., Coelho S.R.G., de Souza R.F., Pero A.C. (2023). Microbial adhesion and biofilm formation by *Candida albicans* on 3D-printed denture base resins. PLoS ONE.

[19] Nayak S.R., Mantri S.S., Nitin S.K., Gupta P., Bhasin A. (2025). Comparative evaluation of surface roughness in CAD/CAM-milled, 3D-printed, and conventional polymethyl methacrylate denture base resin materials: An in-vivo study. J. Clin. Diadn. Res..

[20] Ryu H.S., Lee E.-H., Kwon H.-B., Lim Y.-J., Lee S.-W., Kim M.-J. (2025). The effects of fabrication methods and build orientation on *Candida albicans* adhesion on 3D-printed and conventional denture resin: An in vitro comparative study. BMC Oral Health.

[21] Tseng C.-F., Sung C.-C., Yang Y.-T., Chen F.-N., Mine Y., Chiang Y.-C., Lin Z.-C., Fang M.-L., Chen H.-M., Kok S.-H. (2025). Impacts of surface characteristics on biological responses and biofilm formation of 3D-printed denture base resins: An in vitro study. J. Dent. Sci..

[22] AlBin-Ameer M.A., Alsrheed M.Y., Aldukhi I.A., Matin A., Khan S.Q., Abualsaud R., Gad M.M. (2019). Effect of protective coating on surface properties and *Candida albicans* adhesion to denture base materials. J. Prosthodont..

[23] Hirasawa M., Tsutsumi-Arai C., Takakusaki K., Oya T., Fueki K., Wakabayashi N. (2018). Superhydrophilic co-polymer coatings on denture surfaces reduce *Candida albicans* adhesion—An in vitro study. Arch. Oral Biol..

[24] Teixeira A.B.V., Carvalho-Silva J.M., Ferreira I., Schiavon M.A., Dos Reis A.C. (2024). Silver vanadate nanomaterial incorporated into heat-cured resin and coating in printed resin - Antimicrobial activity in two multi-species biofilms and wettability. J. Dent..

[25] Makvandi P., Nikfarjam N., Sanjani N.S., Qazvini N.T. (2015). Effect of silver nanoparticle on the properties of poly(methyl methacrylate) nanocomposite network made by in situ photoiniferter-mediated photopolymerization. Bull. Mater. Sci..

[26] Aati S., Aneja S., Kassar M., Leung R., Nguyen A., Tran S., Shrestha B., Fawzy A. (2022). Silver-loaded mesoporous silica nanoparticles enhanced the mechanical and antimicrobial properties of 3D printed denture base resin. J. Mech. Behav. Biomed. Mater..

[27] Takamiya A.S., Monteiro D.R., Gorup L.F., Silva E.A., Camargo E.R., Gomes-Filho J.E., de Oliveira S.H.P., Barbosa D.B. (2019). Biocompatible silver nanoparticles incorporated in acrylic resin for dental application inhibit *Candida albicans* biofilm. Mater. Sci. Eng. C.

[28] Bettencourt A.F., Costa J., Ribeiro I.A.C., Goncalves L., Arias-Moliz M.T., Dias J.R., Franco M., Alves N.M., Portugal J., Neves C.B. (2022). Development of a chlorhexidine delivery system based on dental reline acrylic resins. Int. J. Pharm..

[29] Fan C., Chu L., Rawls H.R., Norling B.K., Cardenas H.L., Whang K. (2011). Development of an antimicrobial resin—A pilot study. Dent. Mater..

[30] Durner J., Stojanovic M., Urcan E., Hickel R., Reichl F.-X. (2011). Influence of silver nano-particles on monomer elution from light-cured composites. Dent. Mater..

[31] Yue J., Zhao P., Gerasimov J.Y., van de Lagemaat M., Grotenhuis A., Rustema-Abbing M., van der Mei H.C., Busscher H.J., Herrmann A., Ren Y. (2015). 3D-printable antimicrobial composite resins. Adv. Funct. Mater..

[32] Makvandi P., Jamaledin R., Jabbari M., Nikfarjam N., Borzacchiello A. (2018). Antibacterial quaternary ammonium compounds in dental materials: A systematic review. Dent. Mater..

[33] Jiao Y., Niu L.-N., Ma S., Li J., Tay F.R., Chen J.-H. (2017). Quaternary ammonium-based biomedical materials: State-of-the-art, toxicological aspects and antimicrobial resistance. Prog. Polym. Sci..

[34] Zhang Y., Chen Y., Hu Y., Huang F., Xiao Y. (2018). Quaternary ammonium compounds in dental restorative materials. Dent. Mater. J..

[35] Childs T., Chu L., Barrera L., Ballard C., Fung E., Whang K. (2023). Antimicrobial dental composites with K18-methyl methacrylate and K18-filler. Dent. Mater..

[36] Bapat R.A., Parolia A., Chaubal T., Yang H.J., Kesharwani P., Phaik K.S., Lin S.L., Daood U. (2022). Recent update on applications of quaternary ammonium silane as an antibacterial biomaterial: A novel drug delivery approach in dentistry. Front. Microbiol..

[37] Lee S.-E., Kwon J.-S. (2025). Influence of thermocycling on the antifungal properties of 3D printed denture base material containing zwitterionic and quaternary ammonium compounds. Sci. Rep..

[38] Patel M., Barrera L., Chu L., Whang K. (2024). Development of an antimicrobial, 3D printable denture base material with K18 quaternary ammonium silane-functionalized methyl methacrylate and filler. J. Prosthet. Dent..

[39] Zhou W., Zhao H., Li Z., Huang X. (2022). Autopolymerizing acrylic repair resin containing low concentration of dimethylaminohexadecyl methacrylate to combat saliva-derived bacteria. J. Mater. Sci. Mater. Med..

[40] Liang X., Soderling E., Liu F., He J., Lassila L.V.J., Vallittu P.K. (2014). Optimizing the concentration of quaternary ammonium dimethacrylate monomer in bis-GMA/TEGDMA dental resin system for antibacterial activity and mechanical properties. J. Mater. Sci. Mater. Med..

[41] Han A., Kim K.-M., Kwon J.-S. (2025). Physical/mechanical properties and anti-fungal effects of denture base resin incorporated with cationic quaternary ammonium compounds. Dent. Mater..

[42] Daood U., Matinlinna J.P., Pichika M.R., Mak K.K., Nagendrababu V., Fawzy A.S. (2020). A quaternary ammonium silane antimicrobial triggers bacterial membrane and biofilm destruction. Sci. Rep..

[43] Fitebac K18 Novel Broad-Spectrum Microbicide Antimicrobial Technical Data Sheet. https://fitebactechnology.com/wp-content/uploads/2021/07/K18-Technical-Data-Sheet-AC7.9.21.pdf.

[44] Liu S.-Y., Tonggu L., Niu L.-N., Gong S.-Q., Fan B., Wang L., Zhao J.-H., Huang C., Pashley D.H., Tay F.R. (2016). Antimicrobial activity of a quaternary ammonium methacryloxy silicate-containing acrylic resin: A randomised clinical trial. Sci. Rep..

[45] Gong S.-Q., Epasinghe J., Rueggeberg F.A., Niu L.-N., Mettenberg D., Yiu C.K.Y., Blizzard J.D., Wu C.D., Mao J., Drisko C.L. (2012). An ORMOSIL-containing orthodontic acrylic resin with concomitant improvements in antimicrobial and fracture toughness properties. PLoS ONE.

[46] Gong S.-Q., Niu L.-N., Kemp L.K., Yiu C.K., Ryou H., Qi Y.-P., Blizzard J.D., Nikonov S., Brackett M.G., Messer R.L. (2012). Quaternary ammonium silane-functionalized, methacrylate resin composition with antimicrobial activities and self-repair potential. Acta Biomater..

[47] Gong S.-Q., Epasinghe D.J., Zhou B., Niu L.-N., Kimmerling K.A., Rueggeberg F.A., Yiu C.K., Mao J., Pashley D.H., Tay F.R. (2013). Effect of water-aging on the antimicrobial activities of an ORMOSIL-containing orthodontic acrylic resin. Acta Biomater..

[48] Bellusa S., Chu L., Fung E., Whang K. (2024). Antimicrobial hard denture reliners using quaternary ammonium methacryloxy silicate (K18 QAMS) and K18-functionalized filler. J. Appl. Biomater. Funct. Mater..

[49] Jeon S., Jo Y.-H., Yoon H.-I., Han J.-S. (2022). Effect of phytochemical-filled microcapsules with antifungal activity on material properties and dimensional accuracy of denture base resin for three-dimensional printing. BMC Oral Health.

[50] Sasany R., Jamjoom F.Z., Yilmaz B. (2025). Mechanical and optical properties of additively manufactured denture base resin in different colors modified with antimicrobial substances: An in vitro study. J. Prosthet. Dent..

[51] Fathy S.M., Abdel-Halim M.S., El-Safty S., El-Ganiny A.M. (2023). Evaluation of polymethyl-methacrylate and acetal denture base resins processed by two different techniques before and after nano-chlorohexidine surface treatment. BMC Oral Health.

[52] Sahrir C.D., Lin W.-S., Wang C.-S., Lin H.-E., Wang C.-W., Lin W.-C. (2025). Effects of 3D-printers and manufacturer-specified post-curing units on the dimensional accuracy, compressive strength, and degree of conversion of resin for fixed dental prostheses. J. Dent. Sci..

[53] (2013). Dentistry-Base Polymers-Part1: Denture Base Polymers.

[54] Pinto C.T., Pankowski J.A., Nano F.E. (2017). The anti-microbial effect of food wrap containing beeswax products. J. Microbiol. Biotechnol. Food Sci..

[55] Aati S., Akram Z., Shrestha B., Patel J., Shih B., Shearston K., Ngo H., Fawzy A. (2022). Effect of post-curing light exposure time on the physico-mechanical properties and cytotoxicity of 3D-printed denture base material. Dent. Mater..

[56] Floyd C.J., Dickens S.H. (2006). Network structure of Bis-GMA- and UDMA-based resin systems. Dent. Mater..

[57] Li P., Lambart A.-L., Stawarczyk B., Reymus M., Spintzyk S. (2021). Postpolymerization of a 3D-printed denture base polymer: Impact of post-curing methods on surface characteristics, flexural strength, and cytotoxicity. J. Dent..

[58] Mirizadeh A., Atai M., Ebrahimi S. (2018). Fabrication of denture base materials with antimicrobial properties. J. Prosthet. Dent..

[59] Bettencourt A.F., Neves C.B., de Almeida M.S., Pinheiro L.M., e Oliveira S.A., Lopes L.P., Castro M.F. (2010). Biodegradation of acrylic based resins: A review. Dent. Mater..

[60] Devlin H., Kaushik P. (2005). The effect of water absorption on acrylic surface properties. J. Prosthodont..

[61] Vallittu P., Ruyter I. (1997). The swelling phenomenon of acrylic resin polymer teeth at the interface with denture base polymers. J. Prosthet. Dent..

[62] Han M.S., Seo W.J., Paik H.S., Paik H.S., Hyun J.C., Lee J.W., Kim W.N. (2003). Rheological properties and phase inversion of polypropylene and poly(styrene-co-acrylonitrile) blends. Polym. J..

[63] Adedeji A., Jamieson A.M., Hudson S.D. (1995). Phase inversion in compatibilized immiscible polymer blends with exothermic interfacial mixing. Macromolecules.

[64] Kirby S., Pesun I., Nowakowski A., França R. (2024). Effect of Different Post-Curing Methods on the Degree of Conversion of 3D-Printed Resin for Models in Dentistry. Polymers.

[65] Jang W., Kook G.-S., Kang J.-H., Kim Y., Yun Y., Lee S.-K., Park S.-W., Lim H.-P., Yun K.-D., Park C. (2021). Effect of washing condition on the fracture strength, and the degree of conversion of 3D printing resin. Appl. Sci..

[66] Ling L., Lai T., Malyala R. (2024). Mechanical Properties and Degree of Conversion of a Novel 3D-Printing Model Resin. Polymers.

[67] Al-Qarni F.D., Gad M.M. (2022). Printing accuracy and flexural properties of different 3D-printed denture base resins. Materials.

[68] Liang X., Yu B., Ye L., Lin D., Zhang W., Zhong H.-J., He J. (2024). Recent advances in quaternary ammonium monomers for dental applications. Materials.

[69] Srinivasan M., Chien E.C., Kalberer N., Alambiaga Caravaca A.M.A., Castelleno A.L., Kamnoedboon P., Sauro S., Ozcan M., Muller F., Wismeijer D. (2022). Analysis of the residual monomer content in milled and 3D-printed removable CAD-CAM complete dentures: An in vitro study. J. Dent..

[70] Van Landuyt K.L., Nawrot T., Geebelen B., De Munck J., Snauwaert J., Yoshihara K., Scheers H., Godderis L., Hoet P., Van Meerbeek B. (2011). How much do resin-based dental materials release? A meta-analytical approach. Dent. Mater..

[71] Alansy A.S., Saeed T.A., Guo Y., Yang Y., Liu B., Fan Z. (2022). Antibacterial dental resin composites: A narrative review. Open J. Stomatol..

[72] Jin G., Liu Y., Zhang Z., Yim Y., Lee D.G., Shim M.S., Kim R., Kim J.-E. (2025). Effect of thermal aging on a urethane acrylate-based 3D printing resin incorporated with antibacterial quaternary ammonium methacrylate. J. Mech. Behav. Biomed. Mater..

[73] Lee Y.-S., Mun S.-J., Kang J.-Y., Han S.-Y. (2024). Effect of *Streptococcus mutans* on the autofluorescence of pathogens causing aspiration pneumonia. Photodiagnosis. Photodyn. Ther..

[74] da Silva F.C., Kimpara E.T., Mancini M.N.G., Balducci I., Jorge A.O.C., Koga-Ito C.Y. (2008). Effectiveness of six different disinfectants on removing five microbial species and effects on the topographic characteristics of acrylic resin. J. Prosthodont..

[75] Pereira-Cenci T., Deng D.M., Kraneveld E.A., Manders E.M., Del Bel Cury A.A., Ten Cate J.M., Crielaard W. (2008). The effect of *Streptococcus mutans* and *Candida glabrata* on *Candida albicans* biofilms formed on different surfaces. Arch. Oral Biol..

[76] Yip K.H.K., Smales R.J. (2012). Implications of oral biofilms in medically at risk persons. J. Biomed. Res..

[77] Brown J. (2007). Oral biofilms, periodontitis and pulmonary infections. Oral Dis..

